# Transcriptional deregulation of stress-growth balance in *Nicotiana benthamiana* biofactories producing insect sex pheromones

**DOI:** 10.3389/fpls.2022.941338

**Published:** 2022-10-26

**Authors:** Mojca Juteršek, Marko Petek, Živa Ramšak, Elena Moreno-Giménez, Silvia Gianoglio, Rubén Mateos-Fernández, Diego Orzáez, Kristina Gruden, Špela Baebler

**Affiliations:** ^1^ Department of Biotechnology and Systems Biology, National Institute of Biology, Ljubljana, Slovenia; ^2^ Jožef Stefan International Postgraduate School, Ljubljana, Slovenia; ^3^ Institute for Plant Molecular and Cell Biology (IBMCP), Consejo Superior de Investigaciones Científicas (CSIC), Universidad Politécnica de Valencia (UPV), Valencia, Spain

**Keywords:** *Nicotiana benthamiana*, transcriptomics, plant biotechnology, jasmonic acid, growth-stress tradeoffs, network analysis, growth penalty, insect sex pheromones

## Abstract

Plant biofactories are a promising platform for sustainable production of high-value compounds, among which are insect sex pheromones, a green alternative to conventional insecticides in agriculture. Recently, we have constructed transgenic *Nicotiana benthamiana* plants (“Sexy Plants”, SxP) that successfully produce a blend of moth (Lepidoptera) sex pheromone compounds (*Z*)-11-hexadecen-1-ol and (*Z*)-11-hexadecenyl acetate. However, efficient biosynthesis of sex pheromones resulted in growth and developmental penalty, diminishing the potential for commercial use of SxP in biomanufacturing. To gain insight into the underlying molecular responses, we analysed the whole-genome transcriptome and evaluated it in relation to growth and pheromone production in low- and high-producing transgenic plants of v1.0 and v1.2 SxP lines. In our study, high-producing SxPv1.2 plants accumulated the highest amounts of pheromones but still maintained better growth compared to v1.0 high producers. For an in-depth biological interpretation of the transcriptomic data, we have prepared a comprehensive functional *N. benthamiana* genome annotation as well as gene translations to *Arabidopsis thaliana*, enabling functional information transfer by using Arabidopsis knowledge networks. Differential gene expression analysis, contrasting pheromone producers to wild-type plants, revealed that while only a few genes were differentially regulated in low-producing plants, high-producing plants exhibited vast transcriptional reprogramming. They showed signs of stress-like response, manifested as downregulation of photosynthesis-related genes and significant differences in expression of hormonal signalling and secondary metabolism-related genes, the latter presumably leading to previously reported volatilome changes. Further network analyses confirmed stress-like response with activation of jasmonic acid and downregulation of gibberellic acid signalling, illuminating the possibility that the observed growth penalty was not solely a consequence of a higher metabolic burden imposed upon constitutive expression of a heterologous biosynthetic pathway, but rather the result of signalling pathway perturbation. Our work presents an example of comprehensive transcriptomic analyses of disadvantageous stress signalling in *N. benthamiana* biofactory that could be applied to other bioproduction systems.

## Introduction

Plant biofactories are emerging as a cost-effective, adaptable, fast, scalable, and sustainable platform for biomanufacturing of high value-added compounds, from small molecules to recombinant proteins (Schillberg et al., 2019; Huebbers and Buyel, 2021). Apart from metabolic engineering efforts aimed at improving production of endogenous plant metabolites with applications in health, nutrition, and agriculture (Lu et al., 2016; Mora-Vásquez et al., 2022), the new plant breeding technologies and advancements in synthetic biology are offering a possibility for utilising well-established and robust plant hosts for the production of various non-endogenous compounds (Tschofen et al., 2016; Owen et al., 2017). *Nicotiana benthamiana* has proven to be a versatile chassis with an extensive toolbox that enables rapid development of transgenic plants as well as mass production of desired products, either with transient or with stable transgenic expression systems (Bally et al., 2018; Molina-Hidalgo et al., 2021). Recently reported achievements include the synthesis of taxol intermediates (Li et al., 2019) and production of SARS-CoV-2-related proteins (Diego-Martin et al., 2020), as well as the first approved plant-produced vaccine (Ward et al., 2021).

One group of small molecules whose production has been envisioned and tested in plants are insect sex pheromones (Petkevicius et al., 2020), chemical signals emitted into the air by a member of a species to attract individuals of the same species for mating. They can be used for agricultural insect pest control and have important advantages over conventional broad-spectrum insecticides, such as species specificity, efficiency at lower concentrations, non-toxicity, rapid degradation, and lower odds of resistance (Rizvi et al., 2021). Upon reaching cost-effective yields, pheromone-producing plants could be used for manufacturing in greenhouses or even as living biodispensers grown in fields (Mateos Fernández et al., 2022). So far, most progress has been made in the production of moth (Lepidoptera) pheromones—fatty alcohols, aldehydes, and acetates, whose biosynthetic pathways have been deciphered in many species (reviewed in Petkevicius et al., 2020; Mateos-Fernández et al., 2022).

**Figure 1:**
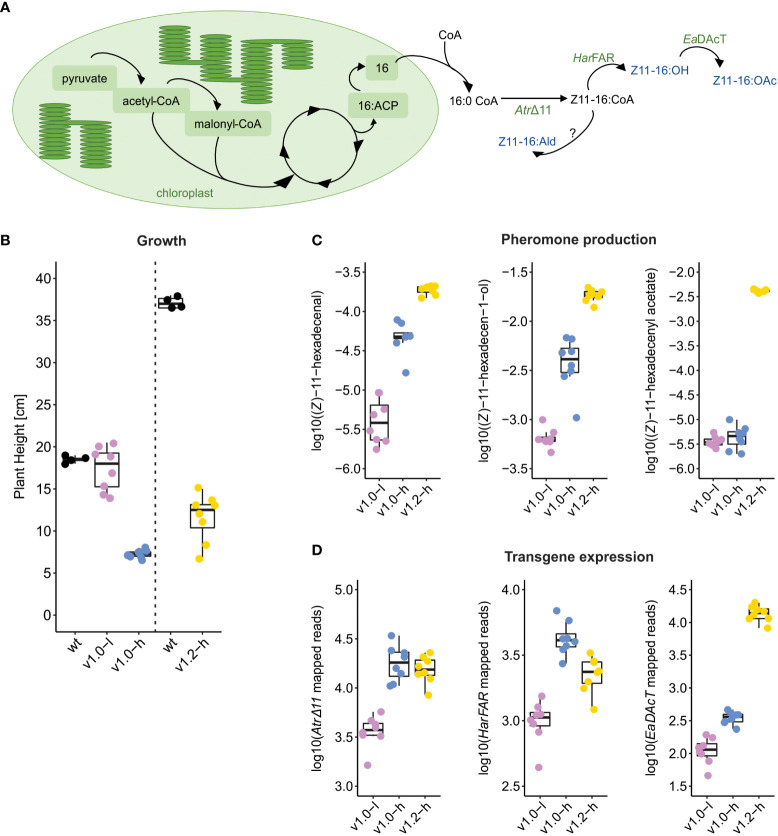
Design and phenotype of SxP. **(A)** Schematic representation of lipid-derived moth pheromone biosynthesis in SxP. Transgene-expressed enzymes are shown in green (*Atr*Δ11, *Har*FAR, and *Ea*DAcT) and the produced pheromones in blue (Z11-16:OH—(*Z*)-11-hexadecen-1-ol, Z11-16:OAc—(*Z*)-11-hexadecenyl acetate, Z11-16:Ald—(*Z*)-11-hexadecenal, resulting from conversion with an unknown endogenous oxidase, as denoted by “?”). **(B–D)** Boxplots of plant height **(B)**, pheromone content **(C)**, and transgene expression **(D)** in SxP and wt *N. benthamiana* plants, selected for RNA-Seq analysis. Plants for sampling of SxPv1.0 and SxPv1.2 were grown in separate experiments, indicated by the dashed vertical line in **(B)**. Pheromone production in SxP ((*Z*)-11-hexadecenal, (*Z*)-11-hexadecen-1-ol, and (*Z*)-11-hexadecenyl acetate) was measured as area under the curve on GC/MS plots, normalised with total ion count (TIC) and log_10_ transformed. Transgene expression (*AtrΔ11* desaturase, *HarFAR* reductase, and *EaDAcT* acetyltransferase) is given as normalised total counts of reads per kilobase of transgene sequence. Sample groups in all boxplots: v1.0-l—SxPv1.0 low producers (purple), v1.0-h—SxPv1.0 high producers (blue), v1.2-h—SxPv1.2 high producers (yellow), N = 8 and wt (black), N=4. Growth and pheromone content for all the grown plants in the experiments are shown in **Supplementary Table S1** and **Supplementary Figure S1**.

Recently, we reported the successful constitutive production of (*Z*)-11-hexadecen-1-ol and (*Z*)-11-hexadecenyl acetate in stable transgenic *N. benthamiana* plants (“Sexy Plants”, SxP), reaching 111.4 and 11.8 μg of product per gram of fresh weight, respectively (Mateos-Fernández et al., 2021). The biosynthesis of insect pheromones is accomplished through a constitutive expression of three genes encoding the *Atr*Δ11 desaturase, the *Har*FAR reductase, and the *Ea*DAcT acetyltransferase (**Figure 1A**). Two transgenic lines were generated: SxPv1.0 contained a truncated *EaDAcT* transgene and therefore produced much lower amounts of (*Z*)-11-hexadecenyl acetate compared with SxPv1.2 plants with a full-length functional *EaDAcT* transgene. Both lines also produced detectable amounts of (*Z*)-11-hexadecenal, presumably as a result of endogenous oxidation of the produced (*Z*)-11-hexadecenyl-CoA. Efficient biosynthesis of sex pheromones resulted in yield-related growth and developmental penalty, diminishing the potential for commercial use of SxP in biomanufacturing (Mateos-Fernández et al., 2021).

Although growth defects associated with constitutive production of specialised metabolites or proteins have been reported in transgenic plants before (e.g., Lu et al., 2017), there is still a lack of insight into the underlying molecular changes, especially at the whole-genome level. To elucidate the perturbations in the SxP production system, we performed RNA-Seq of wild-type and pheromone-producing *N. benthamiana* plants. Differential expression analysis revealed extensive transcriptional reprogramming in high producers with a stress-like signature. In addition, we found a possible activation of the jasmonic acid signalling, leading to suppression of gibberellic acid signalling, which is a known switch between plant growth and stress responses.

## Materials and methods

### Plant growth conditions and sampling

We selected non-transgenic wild-type (wt) and transgenic SxP *N. benthamiana* seeds from two lines of v1.0 low producers (v1.0-low1, v1.0-low2), two lines of v1.0 high producers (v1.0-high1, v1.0-high2), and two lines of v1.2 high producers (v1.2-high1, v1.2-high2; for details on all lines see **Supplementary Table S1** and Mateos-Fernández et al., 2021). SxPv1.0 plants have a truncated *EaDAcT* gene, probably due to an unwanted rearrangement of T-DNA that occurred during the transformation process. Due to the severity of the truncation and the very low production of the acetylated product, the gene product is believed to be non-functional (Mateos-Fernández et al., 2021). Seed germination and plant growth were done as previously described (Mateos-Fernández et al., 2021). Two separate experiments were conducted, where four T_3_ SxPv1.0 lines (two high-producing and two low-producing lines) were grown together with wt plants in the first, and two T_2_ SxPv1.2 lines were grown together with wt plants in the second experiment. Plant height was measured before leaf sampling for pheromone analysis and RNA isolation. Samples were collected from the second and third youngest fully expanded leaves of each plant at the early flowering stage after 35 days of growing in soil, except for high-producing SxPv1.0 lines, which were sampled after 54 days in soil due to their slower development. All samples were frozen in liquid nitrogen immediately after collection and ground afterwards.

### Pheromone analysis

Targeted analysis of pheromone production was performed as previously described (Mateos-Fernández et al., 2021). In short, 50 mg of frozen, ground leaf tissue was weighed in a 10-ml headspace screw-cap vial and stabilised by adding 1 ml of 5 M CaCl_2_ and 150 μl of 500 mM EDTA (pH = 7.5), after which it was sonicated for 5 min. Volatile compounds were captured by headspace solid phase microextraction (HS-SPME). Vials were first incubated at 80°C for 3 min with agitation at 500 rpm. The fibre was then exposed to the headspace of the vial for 20 min under the same conditions of temperature and agitation. Desorption was performed at 250°C for 1 min (splitless mode). Oven conditions started with an initial temperature of 160°C for 2 min, a 7°C min^-1^ ramp until 280°C, and a final hold at 280°C for 6 min. Identification of compounds was performed by the comparison of both retention time and mass spectrum with pure standards (for pheromones) or by comparison between the mass spectrum for each compound with those of the NIST 2017 Mass Spectral Library. Quantification was performed based on measured areas under the curve for pheromone peaks, divided by the total ion count (TIC) of the corresponding sample.

### RNA-Seq and differential expression analysis

Four plant samples per line were selected for RNA isolation and RNA-Seq analysis. RNA was extracted with the GeneJET Plant RNA Purification Mini Kit (Thermo Fisher Scientific, Waltham, MA, USA). Cleaned RNA was DNase treated and quality checked with Bioanalyzer (Agilent, Santa Clara, CA, USA). Preparation of a strand-specific transcriptome library and sequencing on the Illumina HiSeq platform with 150-bp paired-end reads were done by Novogene. Quality control of the reads was performed with FastQC (Andrews, 2010), and quality reports were merged into the final report with MultiQC (Ewels et al., 2016). Mapping with parameters mismatch cost 2, insertion cost 3, deletion cost 3, length fraction 0.9, and similarity fraction 0.9 and read summarisation (counting uniquely mapped paired reads as one) were done in CLC Genomics Workbench 21.0.5 to the *N. benthamiana* reference genome, kindly provided by the *Nicotiana benthamiana* Sequencing Consortium (scaffolding v3.5, annotation v3, downloaded from https://www.nbenth.com/ on 15.12.2021). Differential expression analysis was performed in R, using R packages edgeR and limma (Law et al., 2018) with TMM normalisation. Genes with expression levels below a defined threshold were filtered out before normalisation using the filterByExpr function (min.count = 50 and min.total.count = 100). Experiments with SxPv1.0 and v1.2 plants were analysed separately, contrasting high-producing lines and low-producing lines to wt plants grown for each of the two experiments. Based on quality control (**Supplementary Presentation S1**), three samples (v1.0-wt-1, v1.0-high1-4, and v1.0-high2-4) were excluded from further analyses. Tables with total read counts as well as differential expression analysis results with log_2_ fold change (logFC) and adjusted p-values (pAdj) are deposited at GEO along with raw sequencing reads (series accession GSE192369) and are also available as a supplement to this manuscript (**Supplementary Data Sheet S1**). Transgene expression was determined by mapping Illumina reads to the coding sequences of all three transgenes (see Mateos-Fernández et al. (2021) for description of gene constructs and their sequences) with CLC Genomics Workbench 21.0.5. The obtained total counts were multiplied by the calculated sample TMM normalisation factors to correct for library sizes and divided by gene lengths to obtain normalised counts per kilobase of sequence.

### Functional annotation

Automated functional annotation of gene models from *N. benthamiana* genome v3.5 with MapMan ontology (Thimm et al., 2004) was performed with two versions of the Mercator software: 3.6 (Lohse et al., 2014) and 4v2.0 (Schwacke et al., 2019), mapping to MapMan v.3 and MapMan4 Ontology, respectively. MapMan v.3 ontology was used for generating MapMan mapping and GSEA gene set files using custom Perl scripts. Protein sequences were also submitted to InterProScan using interproscan-5.50-84.0 for annotation with Pfam and InterPro protein families (Jones et al., 2014). For translation between *Arabidopsis thaliana* (ath; Araport 11, Cheng et al., 2017) and *N. benthamiana* (nbe; v3.5) proteins, reciprocal best BLAST (RBB) matches from both directions were determined (ath *vs*. nbe and nbe *vs*. ath). If the match between a particular ath and nbe protein sequence was present in both unfiltered BLAST output result files (from both directions) and if E-value ≤10^-20^ for both directions, the match between the species was kept.

### Gene set enrichment analysis

For Gene Set Enrichment Analysis, we used GSEA 4.2.1 software (Subramanian et al., 2005). RNA-Seq counts were filtered, TMM-normalised (using edgeR package), and transformed to logCPM (log_10_ counts per million). We excluded samples v1.0-wt-1, v1.0-high1-4, and v1.0-high2-4 from GSEA, as explained above. MapMan v.3 BINs were used as gene sets and GSEA was run as previously described (Ramšak et al., 2021).

### Differential VOC abundance

Statistical analysis of non-targeted volatilome data from Mateos-Fernández et al. (2021) was done by comparing log_10_-transformed peak areas measured in arbitrary units (a.u.) and statistical testing with Kruskal–Wallis one-way analysis of variance combined with the pairwise Wilcoxon rank-sum test.

### Visualisation of differentially expressed genes in MapMan and knowledge networks

Differentially expressed genes were visualised in the context of biological pathways and processes in MapMan v3.6.0 using the custom mapping file generated with Mercator 3.6. Differential network visualisations were performed using DiNAR (Zagorščak et al., 2018) utilising two embedded prior knowledge networks: Plant Immune Signalling network and *A. thaliana* Comprehensive Knowledge Network (Ramšak et al., 2018). To translate *N. benthamiana* v3.5 gene models to both *A. thaliana* and *Solanum tuberosum* gene nodes in either of the two networks, we used RBB translation of *N. benthamiana* genes to *A. thaliana* (see above). Since for many gene IDs there was more than one identified homolog, we used a prioritisation script (available at https://github.com/NIB-SI/DiNAR/tree/master/IDprioritization) for choosing the most interesting homolog based on the logFC and pAdj values in our differential expression dataset. Its outputs, used as inputs for DiNAR, are available in **Supplementary Data Sheet S2**.

## Results

SxP are transgenic *N. benthamiana* moth sex pheromone biofactories (**Figure 1A**) with a growth penalty phenotype. To improve their potential as living biodispensers, we were interested in deciphering the underlying molecular changes associated with high production and identifying potential targets for growth optimisation. Therefore, we performed RNA-Seq on leaf samples from high- and low-producing SxP lines of v1.0 and v1.2 as well as wt *N. benthamiana* and analysed them in relation to growth and pheromone content data.

### High-producing SxPv1.2 plants accumulate the highest amounts of pheromones but still maintain better growth compared to v1.0 high producers

Similar to our previous study (Mateos-Fernández et al., 2021), the sampled SxP lines showed different extents of growth penalty (**Figure 1B**; **Supplementary Figure S1**). SxPv1.0 low producers (v1.0-l) had little to no growth penalty, with plant heights at sampling time not differing significantly from wt plants (**Figure 1B**). High producers of v1.0 (v1.0-h) showed the most severe growth penalty with the lowest heights and the biggest developmental delay as they reached the same stage (determined by the number of fully developed leaves) as wt and low-producing plants 19 days later. SxPv1.2 (v1.2-h) plants reached the same developmental stage as wt plants at the time of sampling but were still significantly smaller compared to wt plants. The differences in heights of wt plants grown along each of the SxP versions (**Figure 1B**) could be attributed to different seasons when the experiments were performed (**Supplementary Table S1**); therefore, comparisons were made for each experiment separately.

SxPv1.2 high producers accumulated the highest amounts of all three pheromone compounds (**Figure 1C**; **Supplementary Figure S1**). The highest production of (*Z*)-11-hexadecenyl acetate in SxPv1.2 is in accordance with their genetic background, as they contain a functional and full-length acetyltransferase gene (*EaDAcT*), while SxPv1.0 do not. The truncated *EaDAcT* transcript in SxPv1.0 also seemed to be less stable, as the read counts were substantially lower in v1.0 compared to v1.2 (**Figure 1D**). SxPv1.2 accumulated (*Z*)-11-hexadecen-1-ol at higher levels compared to v1.0 high producers as well, which differs from the results of our first study (Mateos-Fernández et al., 2021), where the (*Z*)-11-hexadecen-1-ol accumulation was lower in SxPv1.2. This could be attributed to the fact that in the previous work, first-generation v1.2 plants were used, while in this study, we sampled and analysed their progeny. Despite the much higher production of (*Z*)-11-hexadecenyl acetate in SxPv1.2, the pool of the precursor (*Z*)-11-hexadecen-1-ol did not seem to be affected, supporting the need for the improvement of the rate-limiting acetyltransferase step (Kallam et al., 2022).

High producers clearly show more severe penalty than low producers, indicating that a certain pheromone production level needs to be reached to trigger growth impairment. However, the extent of growth penalty in high producers cannot be unambiguously linked to the amount of accumulated pheromones, as demonstrated by absence of correlation between plant height and the amount of accumulated pheromones within each high-producing group (**Supplementary Figure S2**), as well as the fact that v1.2 high producers with higher accumulation of all three compounds grow better than v1.0 high producers. This indicates that, besides direct toxicity of produced compounds and/or metabolic limitations, other mechanisms might underlie the observed growth limitation.

### Moth pheromone production rate is reflected in the extent of transcriptional changes

**Figure 2:**
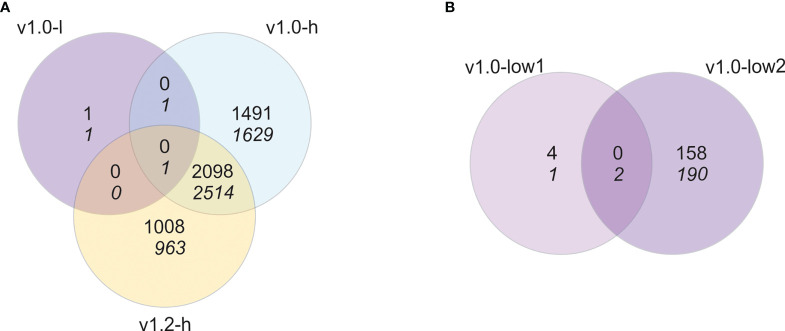
Transcriptional reprogramming is specific for high-producing SxP. Venn diagrams of differentially expressed genes when contrasting **(A)** samples from pheromone producing plants (v1.0-l, v1.0-h, and v1.2-h with N = 8, 6, and 8, respectively) to wt *N. benthamiana* plants or **(B)** samples from the two individual lines of low pheromone-producing plants (v1.0-low1, v1.0-low2, both N = 4) to wt plants. Numbers on the top correspond to upregulated genes and numbers in italic on the bottom to downregulated genes (|logFC| > 1, pAdj < 0.05).

To explore the possible underlying molecular changes causing growth penalty in pheromone-producing lines, we analysed the transcriptomes of SxP and wt plants. Gene expression in all three groups (v1.0-l, v1.0-h, and v1.2-h) was contrasted against the respective wt group for each of the SxP versions. Out of 57,221 genes defined in the *N. benthamiana* v3.5 genome, 31,114 genes were expressed above the threshold in either of the two sampling experiments and were considered for differential expression testing, which gave 9,707 differentially expressed genes (|logFC| > 1, pAdj < 0.05) in at least one of the three comparisons (**Figure 2A**).

The results pointed towards a weak transcriptional response in SxPv1.0 low producers, as we identified only four differentially expressed genes. Nevertheless, the differential expression of genes in each of the low-producing lines in contrast to wt separately resulted in seven differentially expressed genes in v1.0-low1 and 350 differentially expressed genes in the v1.0-low2 line (**Figure 2B**). This difference is a result of the fact that the gene expression profiles of the low1 line were more similar to wt than the low2 line (**Supplementary Figure S3**). Transcriptomic results coincide with pheromone production, as v1.0-low2 accumulated higher levels of (Z)-11-hexadecenal compared to v1.0-low1 (**Supplementary Figure S1**). This indicates a stronger transcriptional response in low producers with higher accumulation of hexadecenal and therefore the potential of a dose-dependent transcriptional response to pheromone production, at least at lower production values.

Transcriptional reprogramming is considerably more extensive in high producers of both v1.0 and v1.2 with 7,734 and 6,584 differentially expressed genes, respectively, out of which 4,613 were differentially expressed in both versions (**Figure 2A**). Transcriptional profiles of high producers of both SxP versions show higher similarity between lines than low producers do (**Supplementary Figure S3**), which might be a consequence of more homogenous pheromone accumulation within each version (**Supplementary Figure S1**). Therefore, we decided to proceed with expression analyses in high producers with samples grouped into SxPv1.0 and SxPv1.2 high producers and not into separate lines.

### Moth pheromone biosynthesis triggers stress-like transcriptional response in high producers

For biological interpretation of the transcriptome data, we generated different *N. benthamiana* gene annotations: MapMan BINs and descriptions according to v.3 and 4 of the ontology, InterPro and Pfam IDs, and closest *A. thaliana* gene matches as determined by the reciprocal best BLAST (RBB) approach. Out of 57,221 genes, we got MapMan ontology annotations (assignment to any BIN, except for BIN 35 “not assigned”) for 32,467 genes with the v.3 and for 25,610 genes with the version 4 of the ontology (**Supplementary Data Sheet S3**). MapMan ontology annotation was used to construct a MapMan mapping file and a GSEA gene set file, which can be used as direct inputs in both applications (**Supplementary Data Sheet S4**, **Supplementary Data Sheet S5**), providing a valuable resource for *N. benthamiana* transcriptomic analyses in various applications.

**Figure 3:**
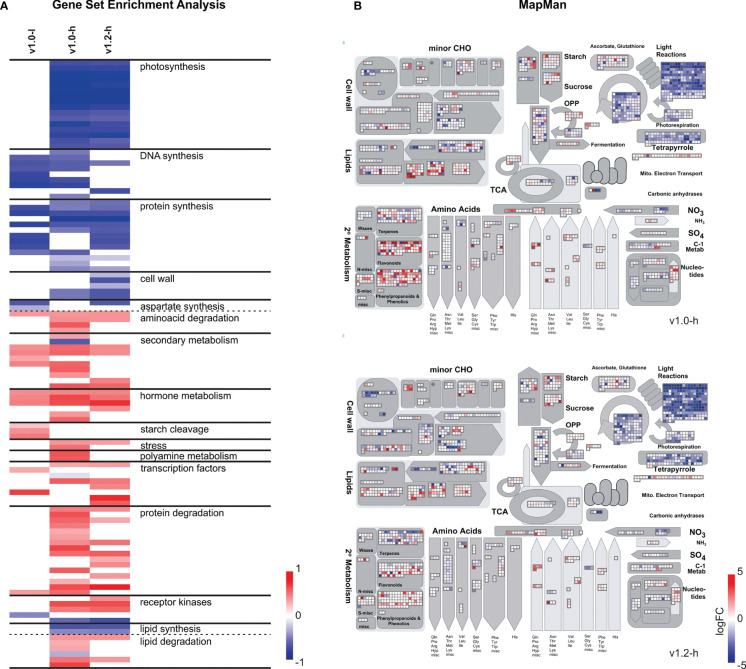
Functional analysis of differentially expressed genes in SxP. **(A)** Gene set enrichment analysis of SxP presented as a heatmap. Gene sets (MapMan BINs) enriched with downregulated genes are shown in blue and gene sets enriched with upregulated genes are shown in red. Contrasting was done for each of the transgenic groups (v1.0-l, v1.0-h, v1.2-h) against wt plants. The colour intensity follows the portion of genes in each gene set that contributed to the enrichment score, as given on the scale at the bottom right of the heatmap. Only selected gene sets with q-value <0.01 are shown, manually grouped into functional clusters given in the figure. A full list of enriched gene sets and gene sets depicted in this figure is presented in **Supplementary Data Sheet S6**. **(B)** MapMan visualisation of metabolism-related differentially expressed genes in SxPv1.0 (top) or SxPv1.2 (bottom) high producers. Only genes with |logFC| > 1 and pAdj < 0.05 in at least one of the comparis ons are displayed. Colour intensities correspond to logFC values (red for positive and blue for negative values) and are given on the scale.

Pathway analysis of gene expression changes in v1.0 and v1.2 high producers revealed transcriptional reprogramming towards stress responses. Gene sets (MapMan BINs) related to photosynthesis, DNA synthesis, protein synthesis, lipid synthesis, and cell wall metabolism were enriched with downregulated genes, while gene sets related to secondary metabolism, hormone metabolism, stress, protein degradation, lipid degradation, and some families of transcription factors showed enrichment with upregulated genes (**Figure 3**; **Supplementary Data Sheet S6**). Although the directionality of response was parallel between the v1.0 and v1.2 producers, some processes did show potentially important differences in magnitude of the response (**Figure 3**; **Supplementary Data Sheet S6**).

Despite of the small number of differentially expressed genes in v1.0 low producers, some pathway changes detected by GSEA, such as enrichment of gene sets related to DNA synthesis and protein synthesis with downregulated genes and of gene sets related to secondary and hormone metabolism with upregulated genes, showed the same pattern as in high producers (**Figure 3A**). Furthermore, starch degradation as well as MADS-box and AUX/IAA transcription factor families were enriched with upregulated genes in low producers but not in high producers (**Supplementary Data Sheet S6**).

### SxP high producers show significant differences in expression of secondary metabolism-related genes, leading to volatilome changes

**Figure 4:**
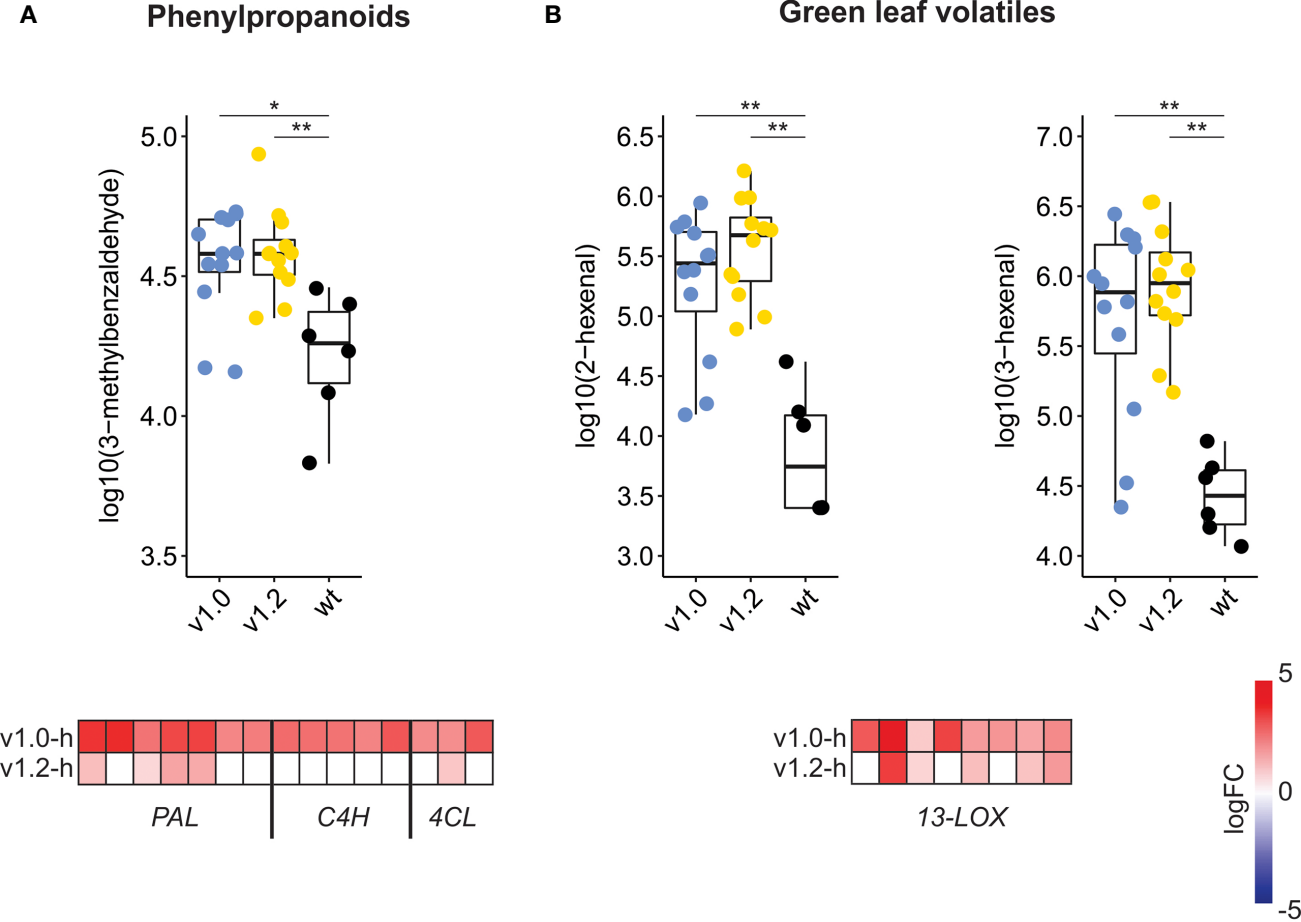
Secondary metabolism changes in SxP on the volatilome and transcriptome levels. **(A)** Abundance of phenylpropanoid-derived 3-methylbenzaldehyde in wt and high-producing SxP and logFC values of phenylpropanoid-related genes (*PAL*, *C4H*, *4CL*), as compared to wt plants. **(B)** Abundance of green leaf volatiles 3- and 2-hexenal in wt and high-producing SxP and logFC values of *13-LOX* genes as compared to wt plants. For volatile compound abundance, ** denotes p < 0.01 and * denotes p < 0.05. Raw data on abundance of volatile compounds were taken from Mateos-Fernández et al. (2021), and analysed data for all metabolites are available in **Supplementary Table S3**; this figure presents only three selected examples of analysed volatiles. Boxplots are shown only for adult samples of v1.0 and v1.2 plants, as described in Mateos-Fernández et al. (2021). Colour saturation denotes logFC values, as given on the scale. Only genes with adjusted p-value < 0.05 are shown. For the full list of depicted genes, see **Supplementary Table S4**.

Functional analyses revealed differences in the extent of transcriptional reprogramming of secondary metabolism-related genes between the two SxP versions, with broader and more intense changes in v1.0 high producers compared to v1.2 high producers (**Figure 3**; **Supplementary Data Sheet S1**), especially in the phenylpropanoid pathway. Homologs of *PAL*, *C4H*, and *4CL* (descriptions and identifiers for all gene short names used in the manuscript are listed in **Supplementary Table S2**), all directing carbon flow towards various branches of phenylpropanoid metabolism, have stronger upregulation in v1.0 high producers (**Figure 4**). To connect these transcriptome changes with metabolite abundance, we have reanalysed previously published volatilome datasets (Mateos-Fernández et al., 2021, **Supplementary Table S3**). Activation of phenylpropanoid biosynthesis on the transcriptomic level correlates with the increase in 3-methylbenzaldehyde abundance in the volatilome of SxP (**Figure 4A**). Higher production of phenylpropanoids through activation of their biosynthesis might therefore also result in increased abundance of phenylpropanoid-derived volatile organic compounds. SxP high producers also produced more green leaf volatiles, oxylipin-derived compounds resulting from the activity of 13-lipoxygenases (13-LOX), whose gene expression was strongly activated in SxP high producers (**Figure 4B**).

### High pheromone production prompts significant reprogramming of hormonal signalling

**Figure 5:**
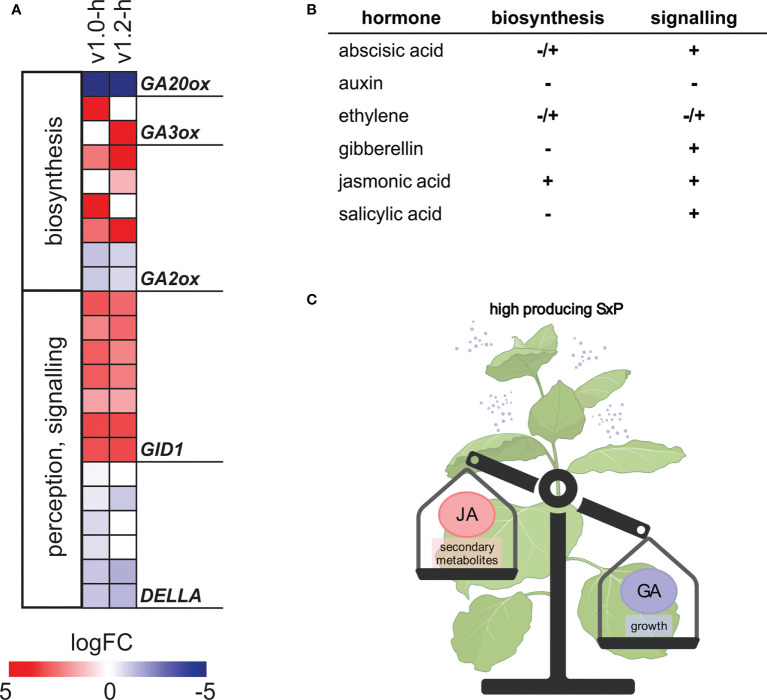
Changes in expression of hormone biosynthesis and signalling genes in SxP high producers. **(A)** Expression of GA-related genes. Colour saturation denotes logFC values, as given on the scale. Only genes with adjusted p-value < 0.05 in at least one group are shown. For the full list of depicted genes, see **Supplementary Table S4**. **(B)** Directionality of hormonal regulation, as summarised from results of differential expression analysis and GSEA (**Supplementary Data Sheet S1**, **Supplementary Data Sheet S6**). – denotes general downregulation, + denotes general upregulation, and +/- denotes changes in both directions. **(C)** Schematic representation of the proposed JA-GA interplay behind SxP growth penalty (created with BioRender.com).

Other pheromone production-related changes evident from transcriptomic data were considerable shifts in expression of hormone metabolism and signalling genes in high producers (**Figure 5B**). Abscisic acid (ABA)-related BINs were enriched in both low and high producers (**Figure 3A**; **Supplementary Data Sheet S6**), with several differentially expressed genes in high producers (**Supplementary Table S4**), indicating activation of ABA signalling. Specifically, ABA receptor subfamilies PYL1/2-like and PYL8/9-like were downregulated and upregulated in high producers, respectively (**Supplementary Table S4**). PYL8/9-like upregulation could indicate higher sensitivity of SxP high producers to growth-inhibiting effects of ABA, since the *pyl8a*, *pyl8b*, and *pyl8c* mutants are insensitive to ABA for growth and development inhibition in shoots (Pizzio et al., 2022). Downstream ABA signalling was activated as well, as evidenced by upregulation of genes with homology to ABA-responsive *ABF2* and *ABF3* transcription factors.

Interestingly, jasmonic acid (JA) biosynthesis and signalling showed a stronger pattern of upregulation in v1.0 compared to v1.2, as was also determined by GSEA (**Supplementary Data Sheet S6**). Gibberellic acid (GA) biosynthesis-related genes showed a distinct pattern of regulation, with strong downregulation of *GA20ox*, encoding a key enzyme in GA biosynthesis, and upregulation of *GA2ox*, *GA3ox*, and *GID1* expression (**Figure 5A**). *GA2ox* homologs with homology to *GA2ox1* and *GA2ox2* had higher expression, while those with homology to *GA2ox9* had lower expression in high-producing SxP. Since GA is a known stimulator of growth in general, deregulation of its biosynthesis and signalling, promoted by JA upregulation, might be a plausible explanation behind the dwarf phenotype of high-producing SxP (**Figure 5C**).

### Jasmonic acid signalling shows consistent upregulation with a stronger response in v1.0 compared to v1.2 high producers

**Figure 6:**
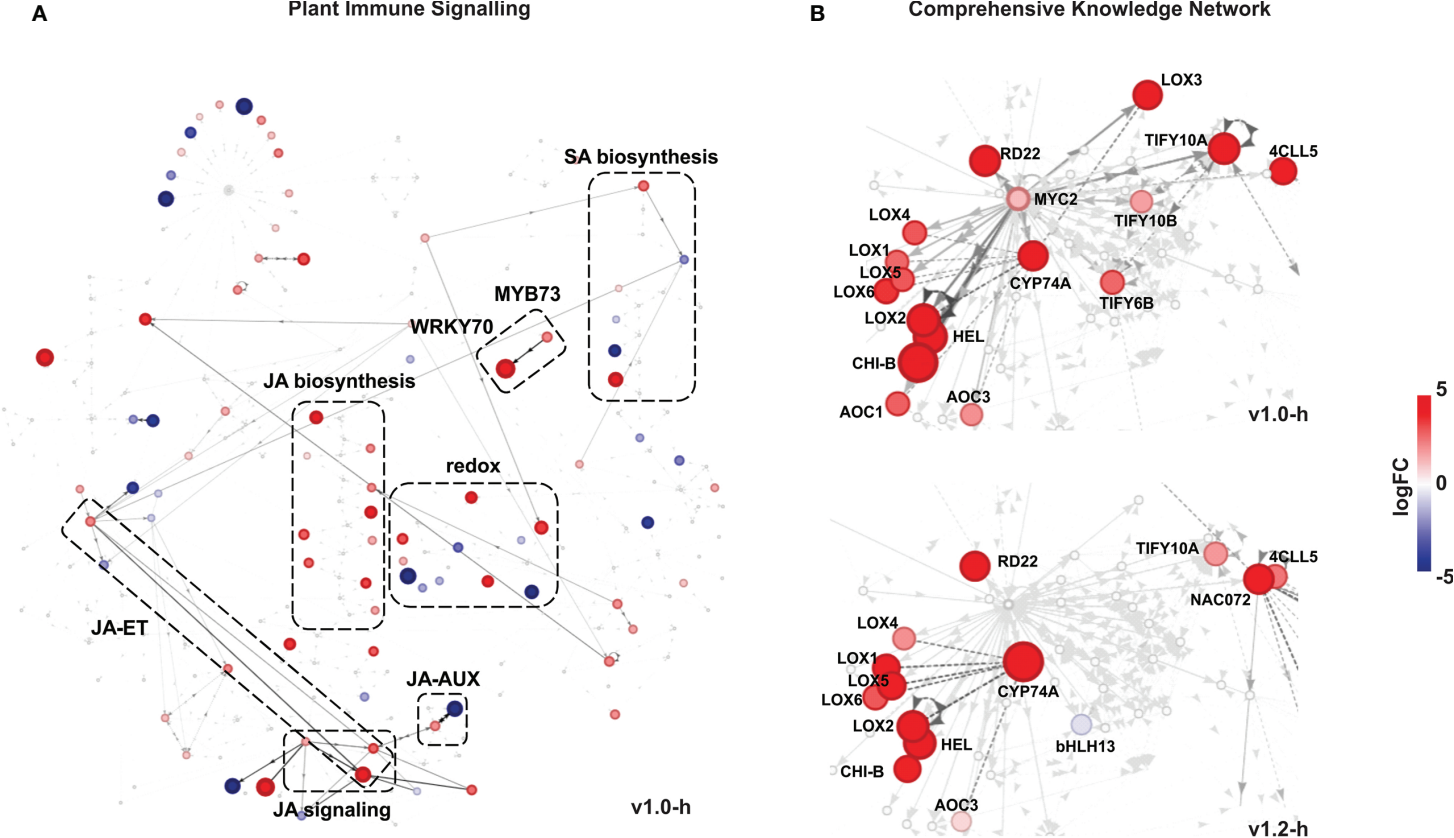
Differentially expressed genes in SxP high producers visualised in knowledge networks. **(A)** Visualisation in the Plant Immune Signalling network with differential expression data for v1.0 high producers (v1.0-h). **(B)** Visualisation of the *MYC2* node and its first neighbours in the Comprehensive Knowledge Network. Differential expression in v1.0 high producers is shown on top and in v1.2 high producers on the bottom. In both networks, only genes with adjusted p-values < 0.05 are shown. When two genes connected with an edge had expression values supported by adjusted p-values < 0.05, the corresponding edge is as shown in darker grey. Colouring and size of nodes denote logFC values, as given on the scale on the right side of the figure. Edges describing the reactions between two nodes are directed and represent activation (full line, unilateral arrows), inhibition (full line), or binding (dashed line, bilateral arrows). Full gene descriptions and identifiers are presented in **Supplementary Table S2**.

Following the results of pathway analyses, which pointed to an orchestrated transcriptional reprogramming of hormonal signalling networks in high-producing SxP, we decided to use knowledge networks of plant gene interactions to further explore active signalling connections and hubs. For that, we have generated homology-based translations of 33,372 *N. benthamiana* genes to *A. thaliana* genes (**Supplementary Data Sheet S3**), enabling usage of differential network analysis with DiNAR (Zagorščak et al., 2018). We visualised differentially expressed genes in the context of two embedded knowledge networks—the Plant Immune Signalling network (PIS) and the *Arabidopsis thaliana* Comprehensive Knowledge Network (CKN) (Ramšak et al., 2018). In the PIS network, which encompasses ethylene (ET), salicylic acid (SA), and JA signalling, the latter showed the most consistent upregulation of all biosynthesis genes, as well as activation of downstream signalling (**Figure 6A**; **Supplementary Data Sheet S2**). Activation of JA signalling was evident also in CKN with the *MYC2* node and its first neighbours showing transcriptional upregulation (**Figure 6B**). The transcriptionally regulated genes included JA biosynthesis genes (*13-LOX*, *AOC*, *4CLL5*, *CYP74A*), biotic stress-related genes (*HEL*, *CHI-B*), and JA signalling genes (*TIFY6B*, *TIFY10A*, *TIFY10B*) upregulated in both v1.0 and v1.2 high producers, with a more prominent activation in v1.0. Active edges in the PIS network also indicate possible interactions between JA signalling and ET (through *EIN3*), as well as SA (through *WRKY70* and *MYB73*) and auxin (AUX) (through *WRKY57*) signalling (**Figure 6A**). *WRKY57* was previously indicated as a defence-enhancing transcriptional regulator involved in the core JA-mediated stress-growth regulatory model in *A. thaliana* (Zhang et al., 2020).

## Discussion

Sustainable production of insect sex pheromones in plant biofactories is currently an emerging topic in plant biotechnology (Xia et al., 2021; Mateos Fernández et al., 2022; Xia et al., 2022). Sexy Plant, a transgenic *N. benthamiana* moth pheromone biofactory, is a bioproduction platform with a constitutive expression of an entire moth sex pheromone biosynthetic pathway, producing and emitting the desired final products (Mateos-Fernández et al., 2021). Induced growth penalty, coinciding with high pheromone production, was recognized as one of the important obstacles in SxP development (Mateos-Fernández et al., 2021). Engineered plants with constitutive transgene expression are often reported to exhibit different deleterious growth and development-related phenotypes, described for production of Pr55^gag^ protein (Scotti et al., 2015), anthocyanins (Fresquet-Corrales et al., 2017), betalain (Polturak et al., 2016), patchoulol (Wu et al., 2006), and squalene (Wu et al., 2012), among others. In astaxanthin-producing tobacco, introduction of inducible, instead of constitutive, production abolished the oxidative stress response and resolved growth penalty (Agrawal et al., 2022), suggesting that change in redox potential was the underlying cause of growth penalty. To gain comprehensive insight into bioproduction-induced growth restrictions in SxP, we have analysed whole-genome transcriptome of transgenic lines with different pheromone production and growth phenotypes.

The transcriptional changes in SxP indicated that the growth penalty was not solely a consequence of a higher metabolic burden imposed by the transgene expression but rather the result of signalling pathways perturbation. The new *N. benthamiana* functional annotations and translations to *A. thaliana* helped us to deduce that vast transcriptomic reprogramming in high producers of both SxP versions might be related to stress or development-induced hormonal changes. We therefore focused on hormonal deregulation as a possible explanation for the growth phenotype. Although several genes related to different plant hormones were differentially regulated (**Figure 5B**), network analyses identified JA to be most consistently and strongly activated both on the level of its biosynthesis and on downstream signalling, evidenced by the activation of the key transcriptional regulator *MYC2* (**Figure 6**). JA is a major regulator of plant growth, defence, and development, whose elevated levels enhance the plant’s defences but also lead to reduced growth (Attaran et al., 2014; Huang et al., 2017; Savchenko et al., 2019). It also activates biosynthesis of different plant secondary metabolites (Baebler et al., 2002; Wasternack and Strnad, 2019), which was observed for the phenylpropanoid pathway in SxP high producers on both the transcriptional and metabolic levels, the latter detected in their volatilome profiles (**Figure 4**).

Interestingly, the *de novo* lipid biosynthesis pathway was transcriptionally downregulated (**Figure 3A**), potentially as a direct response to the increased consumption of C16 fatty acids and by that acetyl-CoA due to pheromone biosynthesis, limiting the availability of acetyl-CoA for other anabolic processes. It is known that *Atr*Δ11 desaturase has a high transformation rate (Ding et al., 2014), supporting the hypothesis of a strong drain imposed on the lipid biosynthesis by pheromone production. Activation of *LOX-13* with concurrent downregulation of fatty acid biosynthesis could limit biosynthesis of other lipid compounds, while providing enough precursors for biosynthesis of stress-related JA and green leaf volatiles.

JA-GA interplay is often predicted to be the main regulator of growth-defence trade-offs (Yang et al., 2012) and has been studied in different plant species, including the *Nicotiana* genus. Previous work of Machado et al. (2017) supports the crucial role of hormonal cross talk as a suppressor of growth and carbohydrate accumulation in *Nicotiana attenuata*. In their experimental system, the JA-GA cross talk seems to be responsible for stunted growth, presumably through downregulation of plant photosynthetic capacity as a direct consequence of reduced GA activity. SxP high producers show a distinct regulatory pattern of GA-related genes, namely, strong downregulation of *GA20ox* and upregulation of *GA3ox*, *GA2ox*, and *GID1* (**Figure 5**), resembling previously described changes in *N. attenuata* mutants with increased JA levels (Heinrich et al., 2013). Similar regulation of GA biosynthesis genes by JA was recently also shown in tomato, with MYC2 inhibiting *GA20ox* and activating *GA2ox* expression (Panda et al., 2022). Based on this evidence, we propose activation of JA signalling with the coordinate GA suppression as a probable molecular mechanism behind pheromone production-induced growth penalty in SxP (**Figure 5C**). As evidenced by deregulation in signalling of other plant hormones (**Figures 5**, **6**), e.g., ABA, there is a possibility of a wider cross talk leading to the final growth phenotype.

Despite the clear pattern of JA-related stress signalling upregulation in SxP, repeatedly seen for two different SxP versions in two separate experiments, and appearing as a plausible molecular perturbation orchestrating the growth arrest, we found limited evidence for the triggering mechanisms. It seems that the transcriptional changes and consequential growth penalty cannot be arising from sex pheromone toxicity or metabolic drainage alone, as this would imply stronger JA deregulation and growth arrest in SxPv1.2 compared to SxPv1.0, which was not the case. There was also no correlation between the measured plant heights and levels of any of the three pheromone compounds within each version of high producers (**Supplementary Figure S2**). Since the versions differ in functionality of the acetyltransferase gene, the changed metabolic flow through the pheromone biosynthetic pathway and consequently the feeding endogenous metabolic pathways might alleviate the stress-inducing potential of pheromone production in SxPv1.2. More severe growth penalty in SxPv1.0 might also be exacerbated by the production of a truncated and non-functional *Ea*DAcT, potentially burdening the protein turnover machinery independently of the pheromone production levels. The differences in the extent of the transcriptional programming between SxPv1.0 and SxPv1.2 could also be just a consequence of positional effects or other factors, since the plants of SxPv1.0 and SxPv1.2 were grown at different times.

The nature of the stress-inducing mechanisms behind pheromone production in general therefore remains to be elucidated. Insect sex pheromones could be a trigger of JA stress response when produced *in planta*, acting as herbivore-associated molecular patterns (Bruce, 2015), with the example of plant defence activation in response to volicitin, a fatty acid–amino acid conjugate present in insect oral secretions (Truitt et al., 2004). However, following this hypothesis, we should expect a stronger response in higher-producing SxPv1.2, which was not the case. Determining the stress-triggering events would require an alternative experimental approach as adult plants with constitutive transgene expression and pheromone production probably represent an already well-established defensive state. To better understand the events prompting the transition from the normal to stressed state and its major regulatory players (Wang and Zhang, 2021), sampling after production initiation in a well-regulated and non-leaky inducible expression system (e.g. Garcia-Perez et al., 2022; Kallam et al., 2022) could give more conclusive insights.

Most work related to the growth-stress dependence has been done in relation to biotic (Brown and Rant, 2013; Ning et al., 2017; da Silva et al., 2019) and abiotic stressors (Bechtold and Field, 2018). Apart from research dedicated to discerning the growth penalty in plants with modified phenylpropanoid and lignin biosynthesis (Reem et al., 2018; Ha et al., 2021; Panda et al., 2021), no major steps have been taken toward understanding molecular mechanisms underlying fitness costs associated with metabolic engineering and/or transgene expression. Results from the analysis of transcriptional reprogramming in SxP support the possible key role of the JA-GA interplay in the insect sex pheromone production-induced growth penalty. In this case, efforts for improving both productivity and growth of SxP biofactories might benefit not only from approaches aiming to optimise metabolic fluxes but also from targeting stress signalling networks. Proof-of-concept work on mutant *A. thaliana* with uncoupled JA and GA signalling has proven that normal plant growth is attainable even in the presence of stress-inducing signals (Campos et al., 2016; Guo et al., 2018) or that severe growth retardation in stress-tolerant plants overexpressing stress-inducible genes can be overcome by co-expressing growth-enhancing genes (Kudo et al., 2019). Targeting orthologous genes in *N. benthamiana* is thus a promising option for establishing a more robust bioproduction chassis. Additionally, controlled regulation of hormonal pathways might even be desired in plants engineered for production of specialised secondary metabolites, for which precursor biosynthesis is hormonally activated (Lu et al., 2016). Considering the goal of developing SxP as living biodispensers in fields, future research should also include assessment of stress-growth signalling interplay in multifactorial settings including different environmental conditions (Kliebenstein, 2016).

Taken together, our work presents an example of comprehensive transcriptomic analyses of yield-reducing stress signalling in plant biomanufacturing that could be applied to other production systems and could in the future contribute to more robust insect pheromone bioproduction without unwanted growth traits.

## Data availability statement

The data presented in the study are deposited in the Gene Expression Omnibus (GEO, https://www.ncbi.nlm.nih.gov/geo/) repository, accession numbers GSE192369, GSE191269 and GSE192368.

## Author contributions

MJ, MP, KG, DO, and ŠB conceived and designed the experiments. MJ, MP, ŽR, EM-G, SG, and RM-F performed the experiments and analysed results. MJ and ŠB drafted the manuscript. All authors contributed to the article and approved the submitted version.

## Funding

This work is part of the SUSPHIRE project (Sustainable Production of Pheromones for Insect Pest Control in Agriculture), a European Research Area Cofund Action ‘ERACoBioTech’, which received funding from the Horizon 2020 research and innovation programme under grant agreement No. 722361. ŠB, KG, MP, ŽR, and MJ received national funding from the Slovenian Ministry of Education, Science and Sport, as well as Slovenian Research Agency’s research core funding grants (P4-0165, P4-0431) and research project grant (Z4-706). EM-G received a PhD grant (FPU18/02019) from the Spanish Ministry of Science, Innovation and Universities. RM-F received a PhD grant (ACIF/2019/226) from Generalitat Valenciana.

## Acknowledgments

*Nicotiana benthamiana* genome data were kindly provided by the *Nicotiana benthamiana* Sequencing Consortium (https://www.nbenth.com/consortium_members.php). We would also like to thank Maja Zagorščak for her advice and help with the DiNAR tool and Víctor García-Carpintero Burgos for help with *N. benthamiana* genome sequences.

## Conflict of interest

The authors declare that the research was conducted in the absence of any commercial or financial relationships that could be construed as a potential conflict of interest.

## Publisher’s note

All claims expressed in this article are solely those of the authors and do not necessarily represent those of their affiliated organizations, or those of the publisher, the editors and the reviewers. Any product that may be evaluated in this article, or claim that may be made by its manufacturer, is not guaranteed or endorsed by the publisher.
