# Supplementary figures and images for: Transcriptional deregulation of stress-growth balance in Nicotiana benthamiana biofactories producing insect sex pheromones
Source: Front Plant Sci. 2022 Oct 26;13:941338. doi: 10.3389/fpls.2022.941338 (PMC9645294; doi:10.3389/fpls.2022.941338)

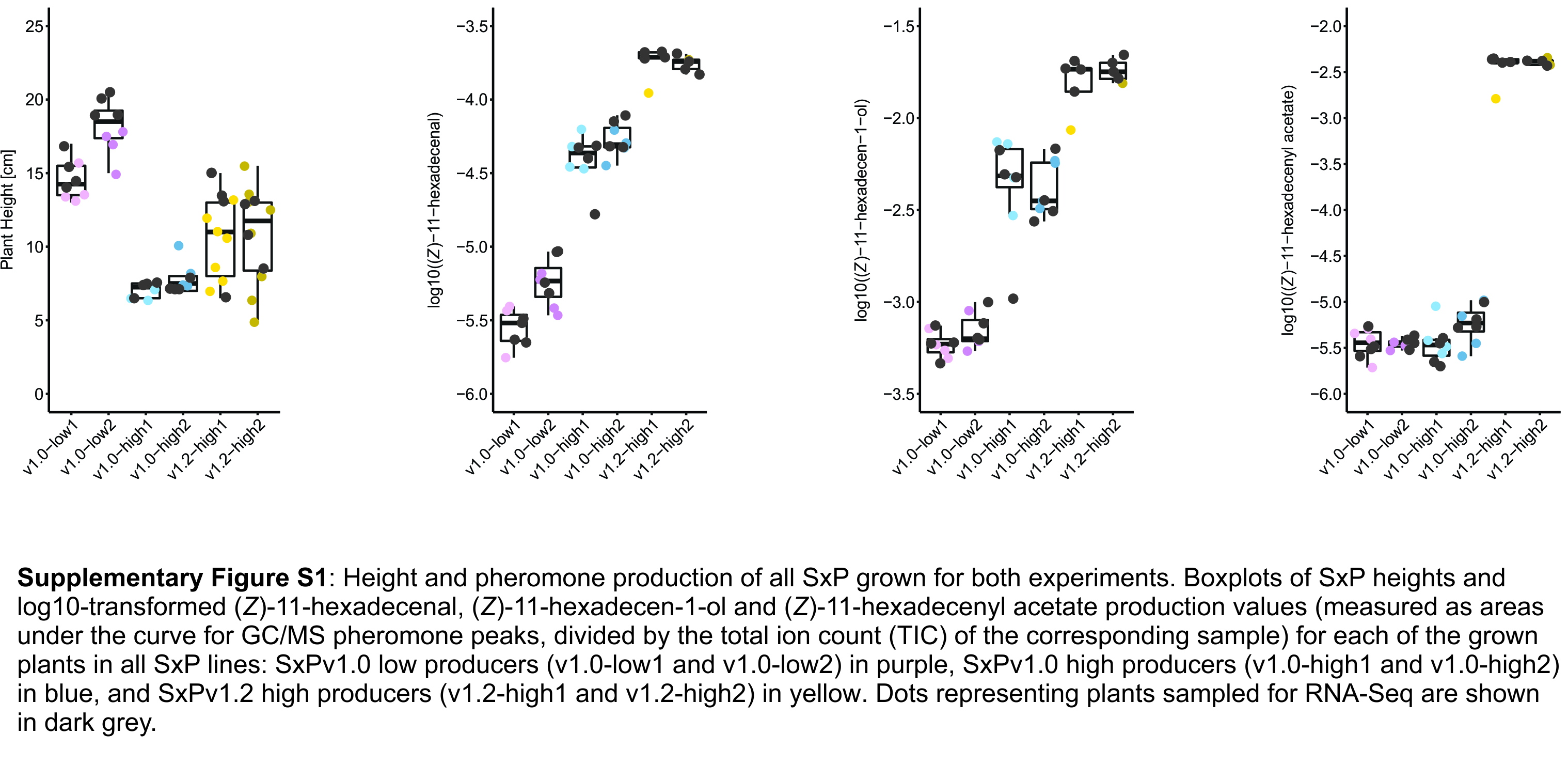

Supplement: Supplementary file 1 [file Image_1.jpeg]

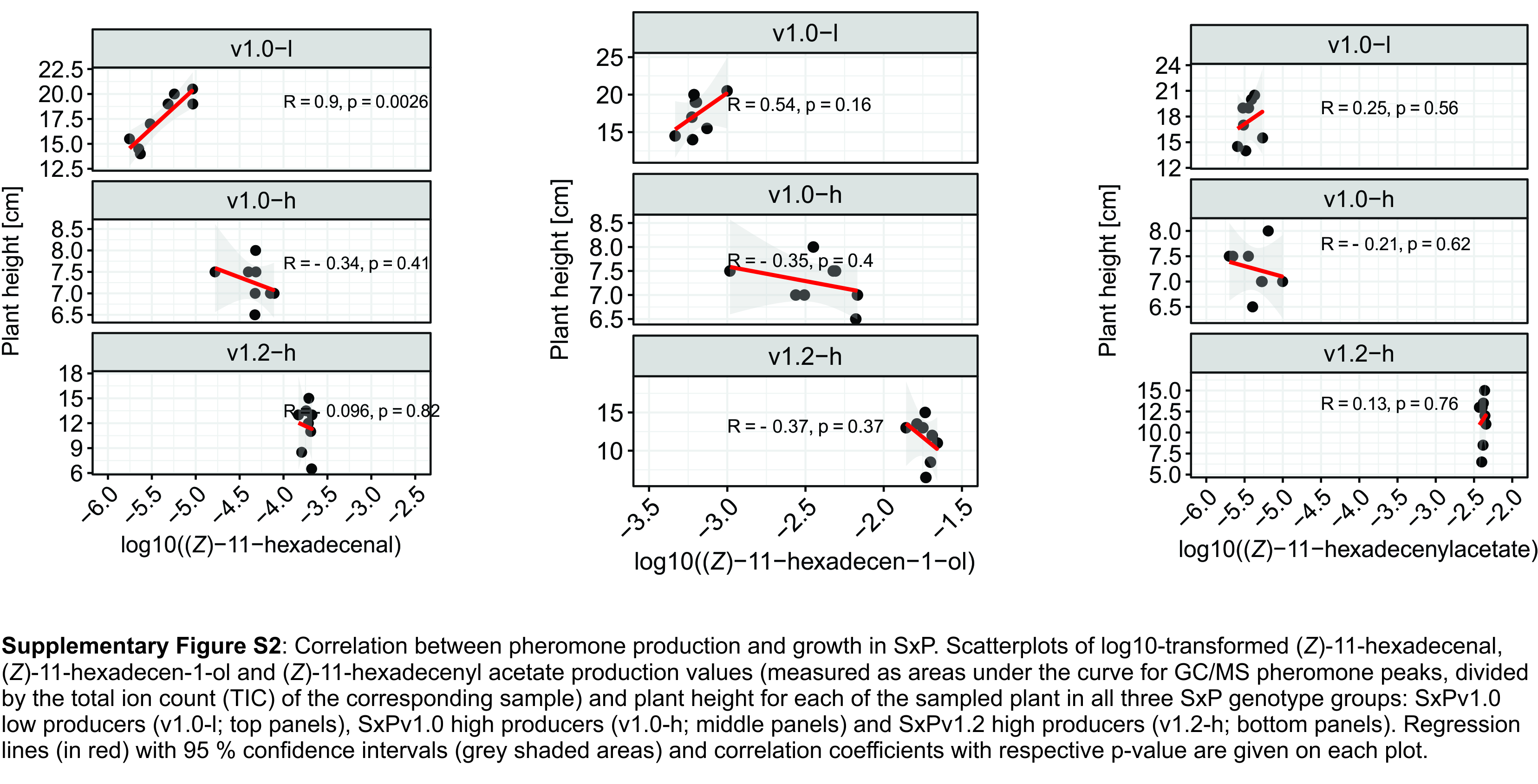

Supplement: Supplementary file 2 [file Image_2.jpeg]

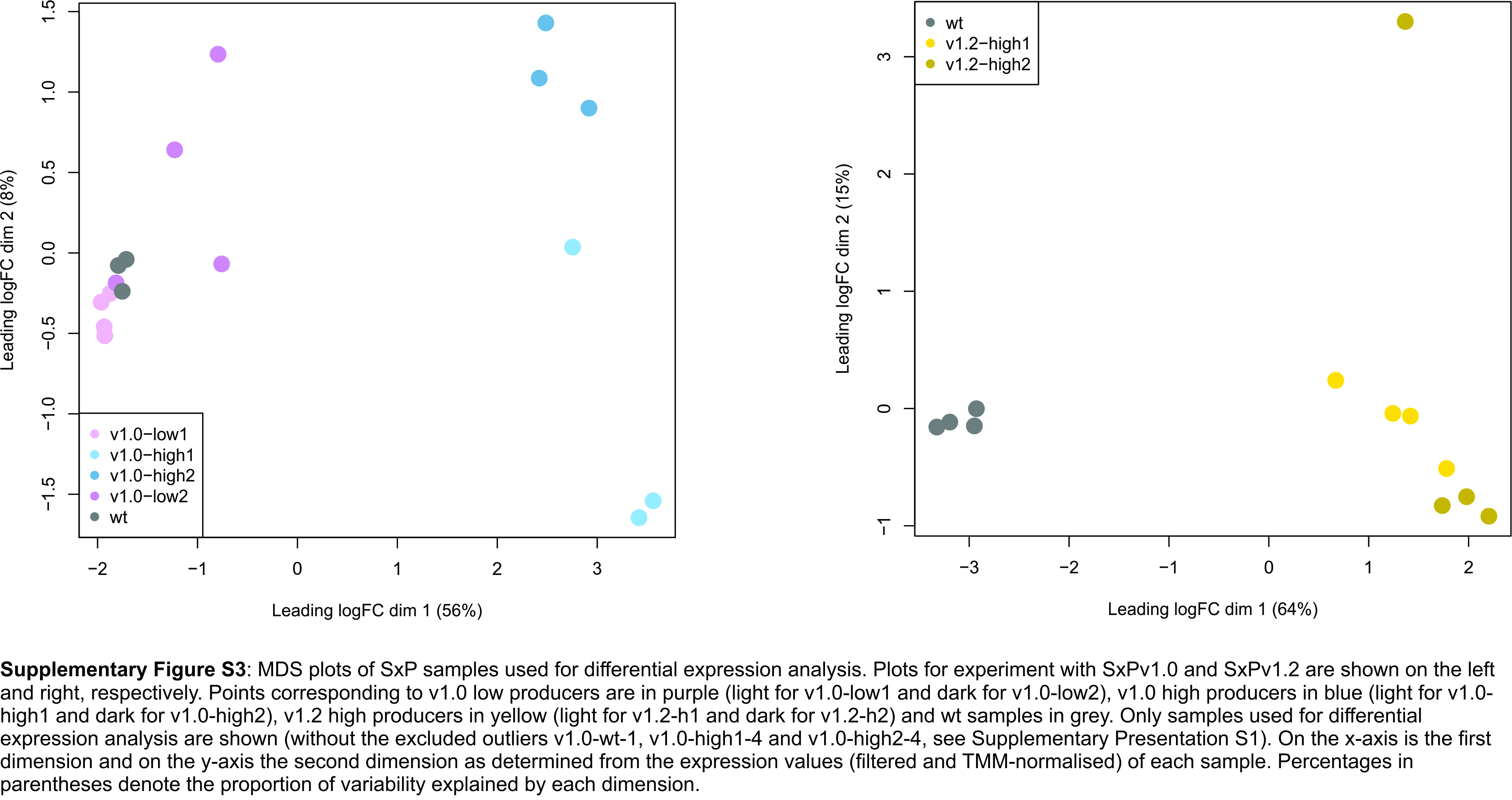

Supplement: Supplementary file 3 [file Image_3.jpeg]
