# Supplementary material for: Transcriptional deregulation of stress-growth balance in Nicotiana benthamiana biofactories producing insect sex pheromones
Source: Front Plant Sci. 2022 Oct 26;13:941338. doi: 10.3389/fpls.2022.941338 (PMC9645294; doi:10.3389/fpls.2022.941338)
Supplement: Supplementary file 14 [file Presentation_1.pptx]

## Slide 1
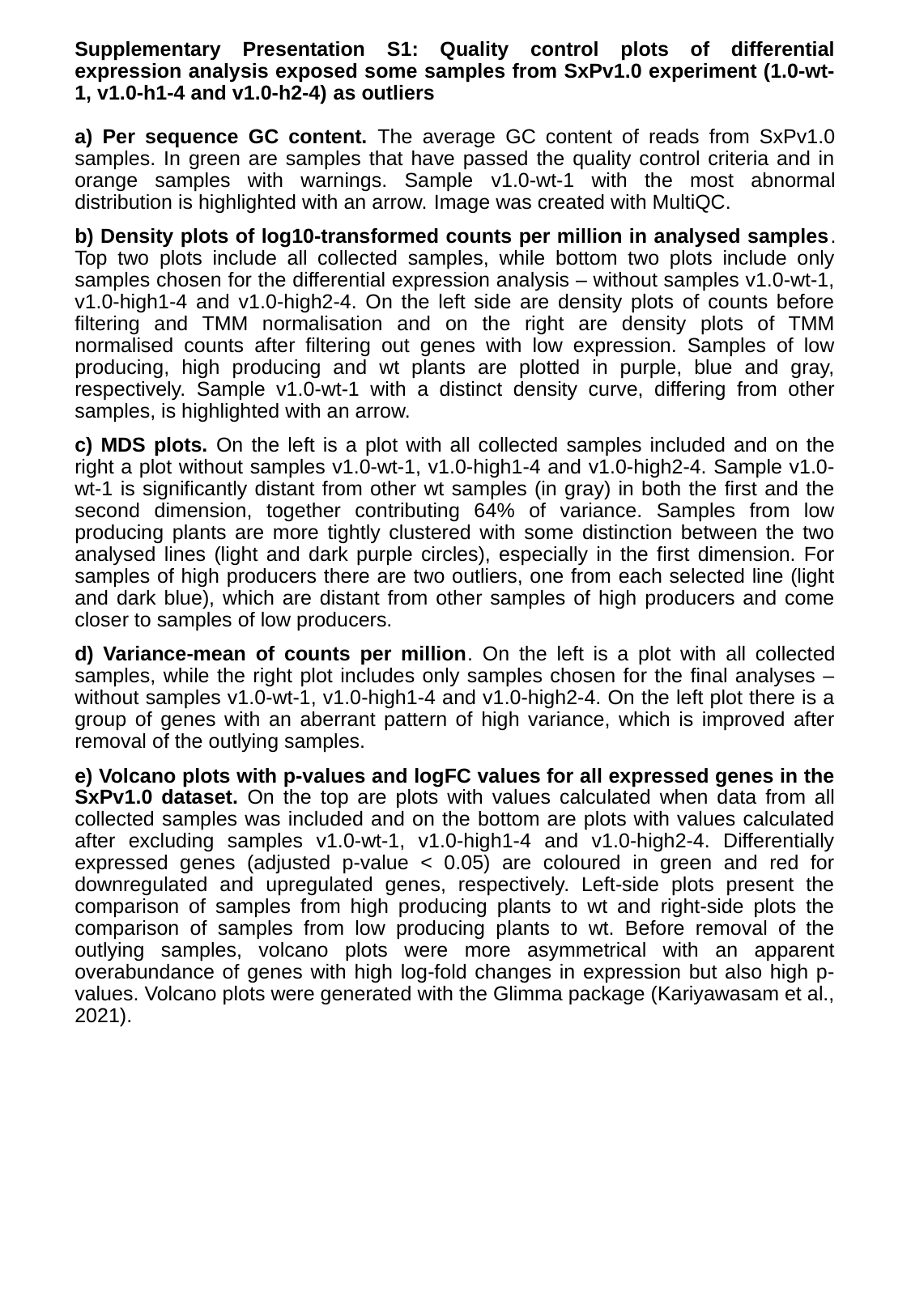

Supplementary Presentation S1: Quality control plots of differential expression analysis exposed some samples from SxPv1.0 experiment (1.0-wt-1, v1.0-h1-4 and v1.0-h2-4) as outliers
a) Per sequence GC content. The average GC content of reads from SxPv1.0 samples. In green are samples that have passed the quality control criteria and in orange samples with warnings. Sample v1.0-wt-1 with the most abnormal distribution is highlighted with an arrow. Image was created with MultiQC.
b) Density plots of log10-transformed counts per million in analysed samples. Top two plots include all collected samples, while bottom two plots include only samples chosen for the differential expression analysis – without samples v1.0-wt-1, v1.0-high1-4 and v1.0-high2-4. On the left side are density plots of counts before filtering and TMM normalisation and on the right are density plots of TMM normalised counts after filtering out genes with low expression. Samples of low producing, high producing and wt plants are plotted in purple, blue and gray, respectively. Sample v1.0-wt-1 with a distinct density curve, differing from other samples, is highlighted with an arrow.
c) MDS plots. On the left is a plot with all collected samples included and on the right a plot without samples v1.0-wt-1, v1.0-high1-4 and v1.0-high2-4. Sample v1.0-wt-1 is significantly distant from other wt samples (in gray) in both the first and the second dimension, together contributing 64% of variance. Samples from low producing plants are more tightly clustered with some distinction between the two analysed lines (light and dark purple circles), especially in the first dimension. For samples of high producers there are two outliers, one from each selected line (light and dark blue), which are distant from other samples of high producers and come closer to samples of low producers.
d) Variance-mean of counts per million. On the left is a plot with all collected samples, while the right plot includes only samples chosen for the final analyses – without samples v1.0-wt-1, v1.0-high1-4 and v1.0-high2-4. On the left plot there is a group of genes with an aberrant pattern of high variance, which is improved after removal of the outlying samples.
e) Volcano plots with p-values and logFC values for all expressed genes in the SxPv1.0 dataset. On the top are plots with values calculated when data from all collected samples was included and on the bottom are plots with values calculated after excluding samples v1.0-wt-1, v1.0-high1-4 and v1.0-high2-4. Differentially expressed genes (adjusted p-value < 0.05) are coloured in green and red for downregulated and upregulated genes, respectively. Left-side plots present the comparison of samples from high producing plants to wt and right-side plots the comparison of samples from low producing plants to wt. Before removal of the outlying samples, volcano plots were more asymmetrical with an apparent overabundance of genes with high log-fold changes in expression but also high p-values. Volcano plots were generated with the Glimma package (Kariyawasam et al., 2021).

## Slide 2
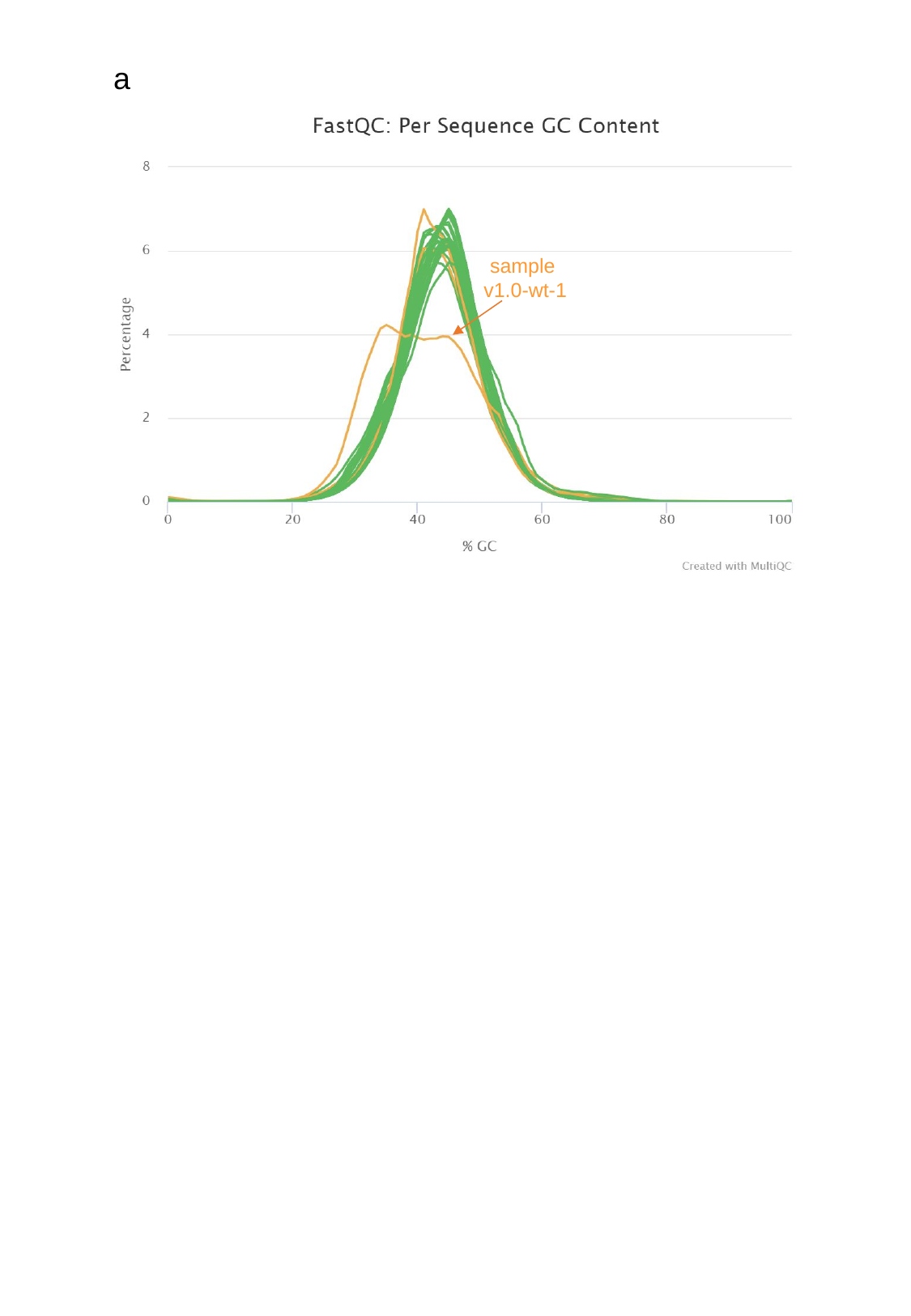

a
sample
v1.0-wt-1

## Slide 3
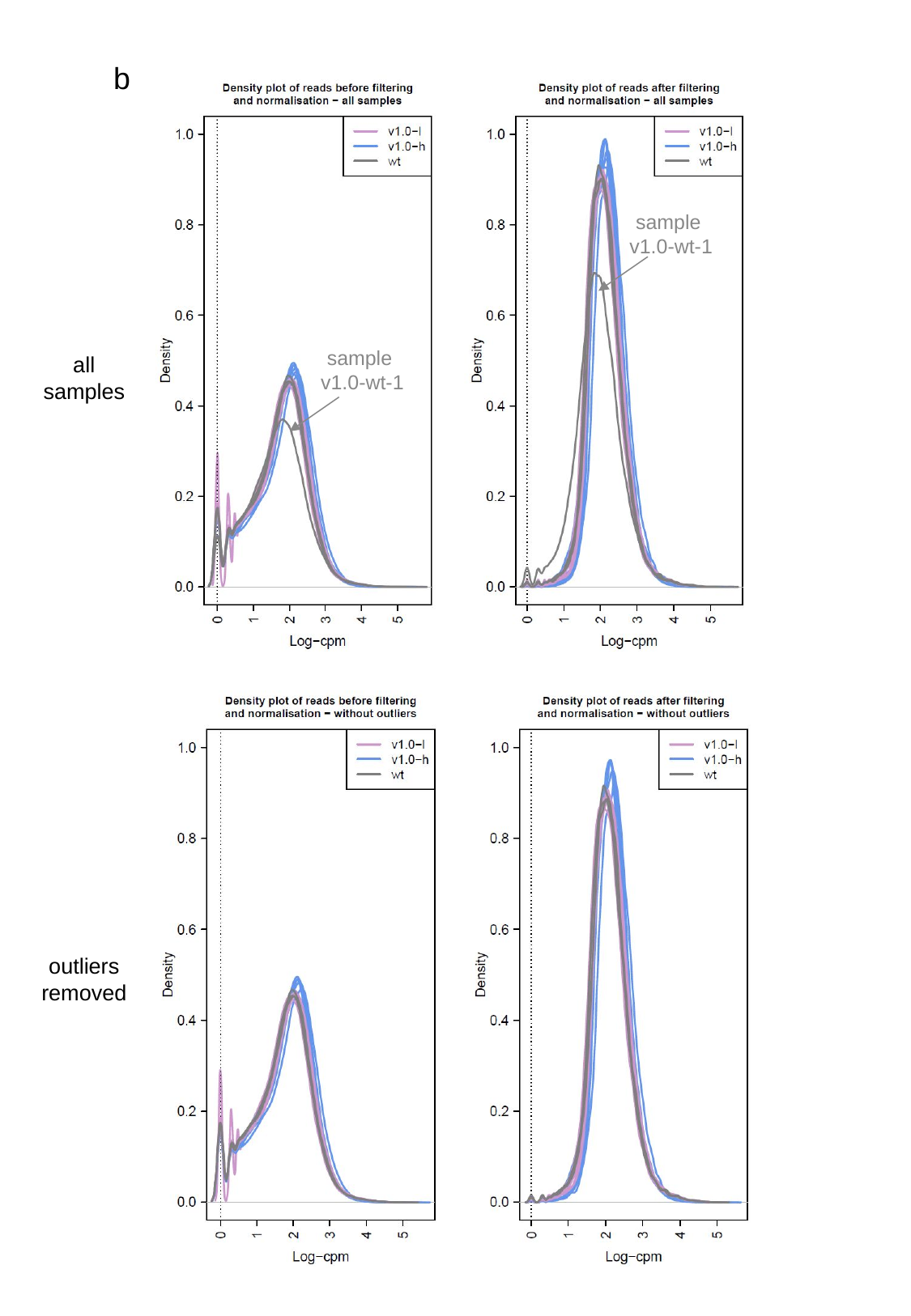

b
sample
v1.0-wt-1
all samples
sample
v1.0-wt-1
outliers removed

## Slide 4
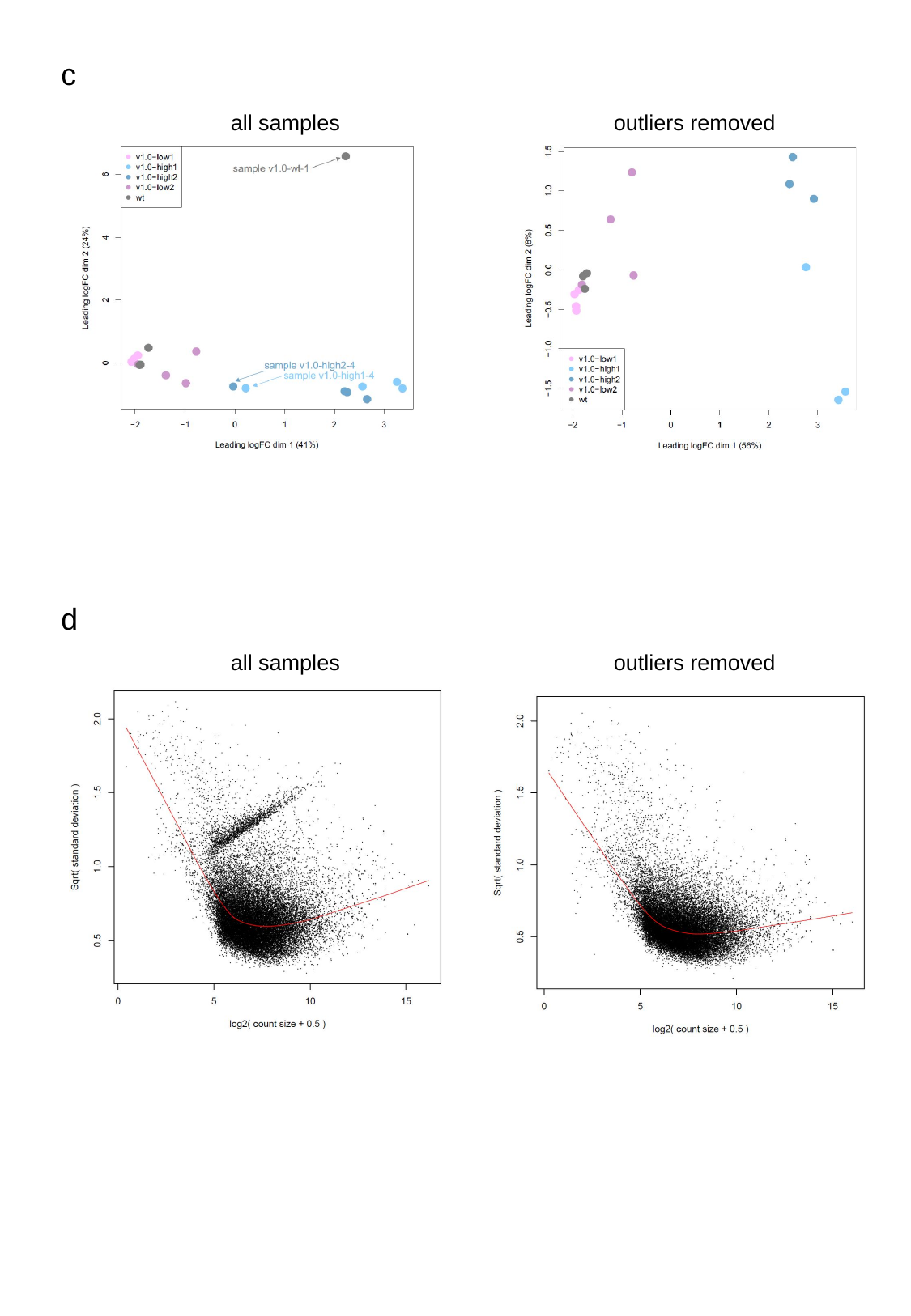

c
all samples
outliers removed
d
all samples
outliers removed

## Slide 5
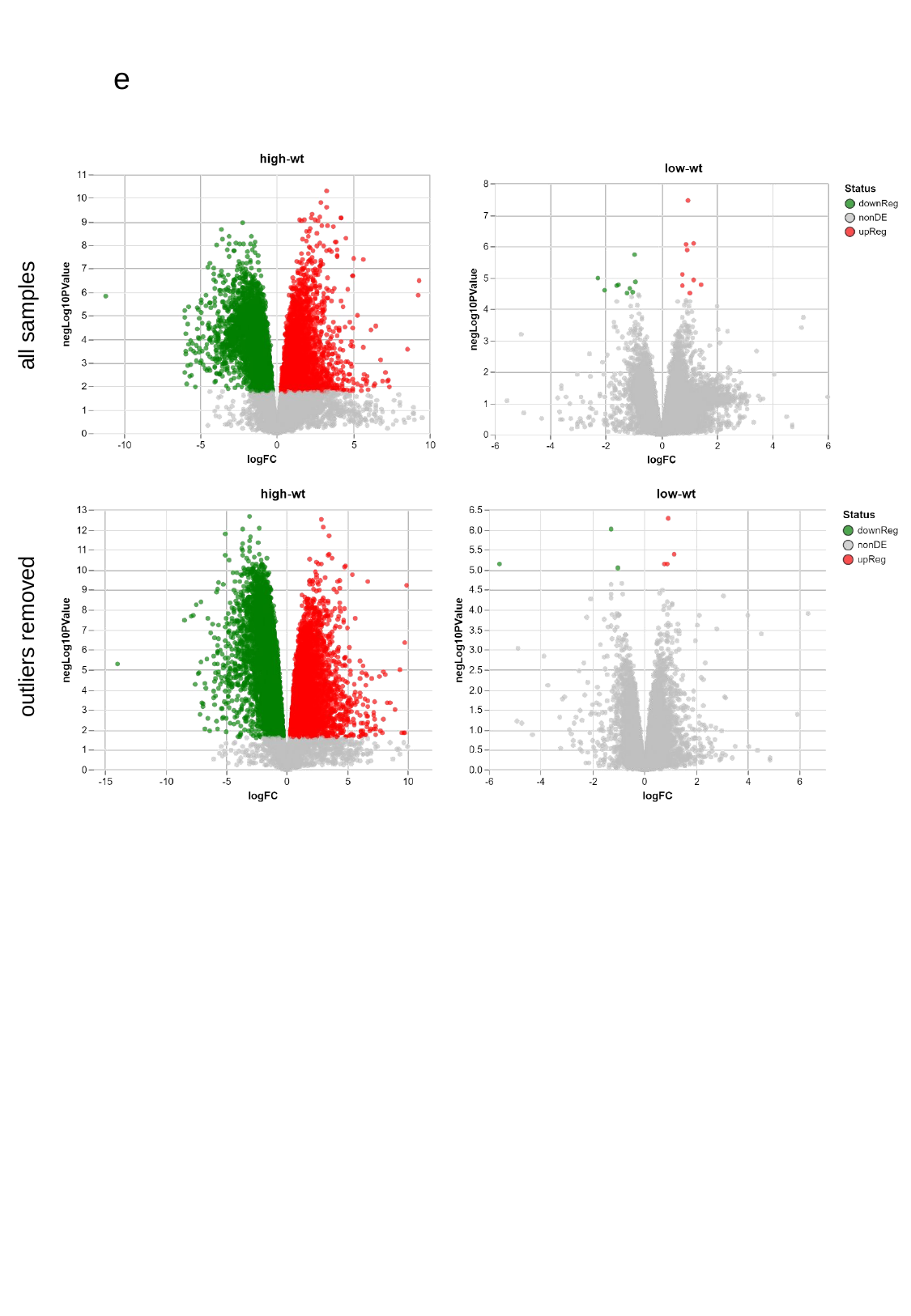

e
all samples
outliers removed

## Slide 6
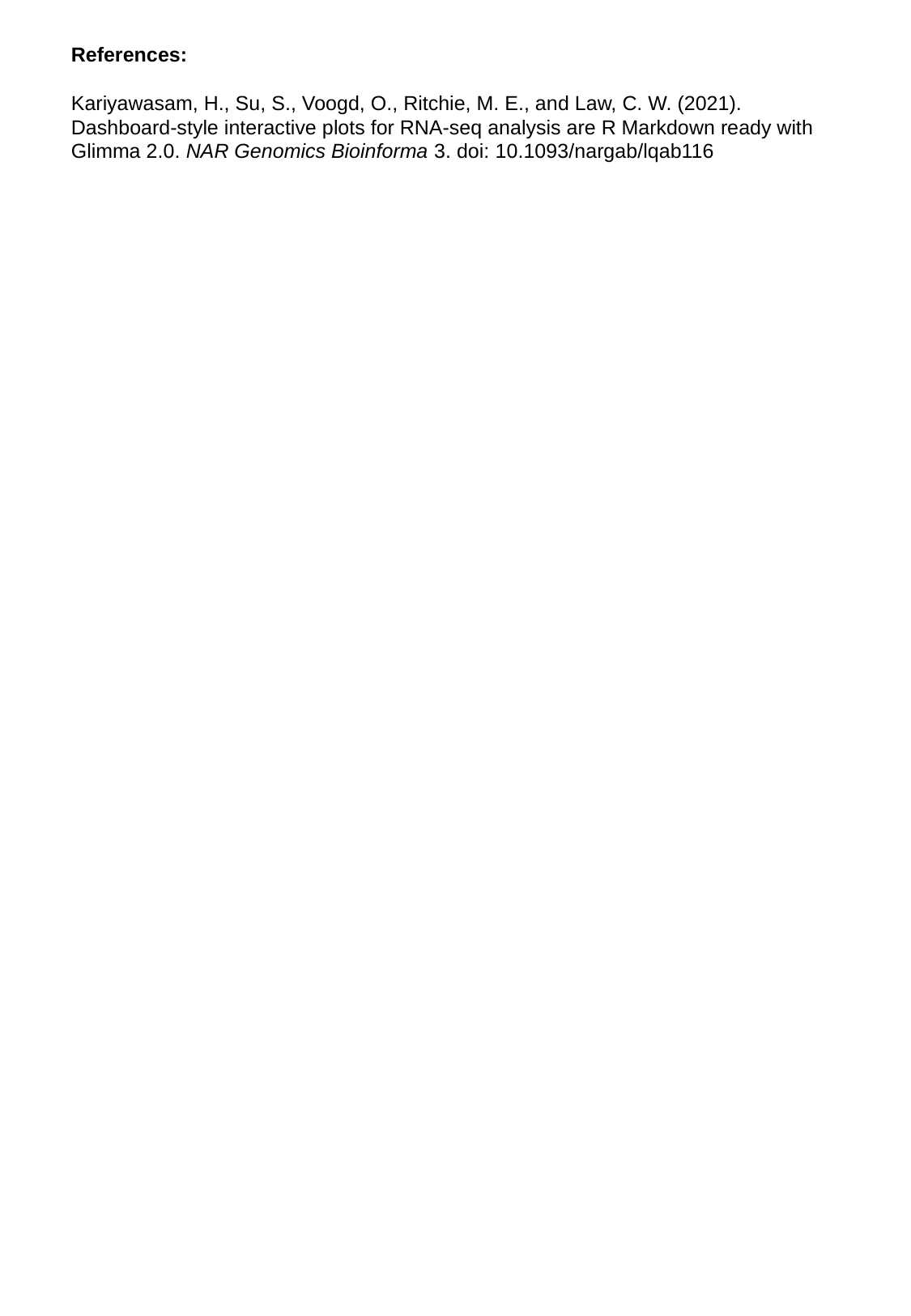

References:
Kariyawasam, H., Su, S., Voogd, O., Ritchie, M. E., and Law, C. W. (2021). Dashboard-style interactive plots for RNA-seq analysis are R Markdown ready with Glimma 2.0. NAR Genomics Bioinforma 3. doi: 10.1093/nargab/lqab116
